# Using Discursis to enhance the qualitative analysis of hospital pharmacist-patient interactions

**DOI:** 10.1371/journal.pone.0197288

**Published:** 2018-05-22

**Authors:** Bernadette A. M. Chevalier, Bernadette M. Watson, Michael A. Barras, William N. Cottrell, Daniel J. Angus

**Affiliations:** 1 School of Pharmacy, The University of Queensland, Brisbane, Queensland, Australia; 2 Department of English, The Hong Kong Polytechnic University, Hung Hom, Kowloon, Hong Kong; 3 Pharmacy Department, Princess Alexandra Hospital, Brisbane, Queensland, Australia; 4 School of Communication and Arts, The University of Queensland, Brisbane, Queensland, Australia; Geisinger Medical Center, UNITED STATES

## Abstract

**Introduction:**

Pharmacist-patient communication during medication counselling has been successfully investigated using Communication Accommodation Theory (CAT). Communication researchers in other healthcare professions have utilised Discursis software as an adjunct to their manual qualitative analysis processes. Discursis provides a visual, chronological representation of communication exchanges and identifies patterns of interactant engagement.

**Aim:**

The aim of this study was to describe how Discursis software was used to enhance previously conducted qualitative analysis of pharmacist-patient interactions (by visualising pharmacist-patient speech patterns, episodes of engagement, and identifying CAT strategies employed by pharmacists within these episodes).

**Methods:**

Visual plots from 48 transcribed audio recordings of pharmacist-patient exchanges were generated by Discursis. Representative plots were selected to show moderate-high and low- level speaker engagement. Details of engagement were investigated for pharmacist application of CAT strategies (approximation, interpretability, discourse management, emotional expression, and interpersonal control).

**Results:**

Discursis plots allowed for identification of distinct patterns occurring within pharmacist-patient exchanges. Moderate-high pharmacist-patient engagement was characterised by multiple off-diagonal squares while alternating single coloured squares depicted low engagement. Engagement episodes were associated with multiple CAT strategies such as discourse management (open-ended questions). Patterns reflecting pharmacist or patient speaker dominance were dependant on clinical setting.

**Discussion and conclusions:**

Discursis analysis of pharmacist-patient interactions, a novel application of the technology in health communication, was found to be an effective visualisation tool to pin-point episodes for CAT analysis. Discursis has numerous practical and theoretical applications for future health communication research and training. Researchers can use the software to support qualitative analysis where large data sets can be quickly reviewed to identify key areas for concentrated analysis. Because Discursis plots are easily generated from audio recorded transcripts, they are conducive as teaching tools for both students and practitioners to assess and develop their communication skills.

## Introduction

All healthcare professionals including hospital pharmacists must possess effective communication skills to ensure they provide high quality patient care [1]. Many hospital pharmacists routinely interact with patients as part of their clinical role within a healthcare team [2–6]. Pharmacists often meet with patients to discuss their medications at transition points of their hospital journey, such as on admission, transfer between wards or discharge from hospital. These transitions have been identified as times when patients are at a higher risk of experiencing medication errors and adverse events [2,7,8]. Transitions are key times for pharmacists to address patients’ concerns about their therapy, review patients’ medications and discuss any changes taking place during their hospital stay [2–6]. Failure by a hospital pharmacist to communicate effectively with patients may negatively impact a patient’s confidence and ability to manage their medications contributing to medication non-adherence [9–12]. Therefore, it is imperative that hospital pharmacists communicate effectively with patients and their caregivers. However, communication taking place between hospital pharmacists and patients is poorly understood with few publications providing little detail about what makes these conversations effective [13–19]. In addition, most hospital pharmacist-patient communication literature is atheoretical [14–20].

To address the atheoretical gap in the literature, we have previously investigated hospital pharmacist-patient exchanges during medication counselling by invoking Communication Accommodation Theory (CAT) as the theoretical framework to analyse and interpret the conversations [21]. This qualitative study used audio recordings and field observations of pharmacist-patient interactions to investigate how well hospital pharmacists utilised CAT strategies in their interactions with patients [21].

CAT is a widely used theoretical framework in healthcare communication research [22–26] and describes the emotional, behavioural and motivational processes underlying communication exchanges [27]. CAT describes communication as being either accommodative or non-accommodative [28]. Accommodation takes place when speakers adjust the way they communicate to bring themselves closer linguistically to the other person. On the other hand, non-accommodative behaviour creates communication barriers between speakers and increases linguistic distance [29].

CAT proposes there are five strategies (approximation, interpretability, emotional expression, discourse management, and interpersonal control) that can be measured to establish the presence or lack of effective communication [28, 30–35]. These are presented in Table 1.

**Table 1:** The five CAT communication strategies.

CAT strategy	Description of strategy	Example of accommodative strategy use
Approximation	Related to speech production where one speaker matches anothers’ dialect/slang or accent, tone, rate of speech or same-saying (repetition of the previous speaker’s words) [32].	Pharmacist slows down or increases their usual speech rate to match that of a patient’s so that the patient understands the information provided about their medications.
Interpretability	Focus on communication competence where speakers adjust the language used and words chosen in their speech to make it easier for the other person to understand them [28].	Pharmacist explains how a medication works to a patient using non-medical, easily understood language.
Discourse management	Involves communication processes to promote conversations and speaker engagement by by asking open-ended questions, demonstrating active listening skills, paying attention to non-verbal cues, and using conversational maintenance such as back-channelling (“hmm”, “yeah”) or repair such as face-maintenance (allowing patients to “save face”) [33].	Pharmacist asks open-ended questions to elicit information from a patient about how well they are tolerating their new prescription.
Emotional expression	Related to how one speaker responds to the other speaker’s emotional needs [31].	Pharmacists accurately assess and respond to patients’ need for reassurance and empathy.
Interpersonal control	Refers to how speakers use their power to exert their own social or professional role in conversations with others [30].	Pharmacists promote equality between themselves and patients through shared decision making.

In our previously reported study, we found that most pharmacists effectively used all five CAT communication strategies during medication counselling by accommodating to patients’ conversational needs. Non-accommodation occurred when pharmacists spoke too quickly, used terms not understood by patients, and did not include patients’ input at the start of the conversation, the agenda-setting phase [21].

Our next step was to use Discursis software to visualise these pharmacist-patient conversations and identify key patterns within the software output that might enhance our previously conducted qualitative analysis. Discursis is a computational analysis support tool developed to assist researchers in analysing communication data. Since its inception, it has been used across many different conversational contexts to help analysts identify turn-taking and engagement patterns. From a transcript of a conversation, Discursis can produce a visual plot which represents the pattern of exchange between speakers in chronological sequence [36–38].

The Discursis analysis of pharmacist-patient interactions presented here will be a novel application of this technology. Discursis software may help identify patterns of effective communication between pharmacists and patients through visual representation of these exchanges. It is important to note that Discursis will be used as an analytical support tool, as it is not intended to replace qualitative analysis.

The aim of this study was to describe how Discursis could be used to enhance qualitative analysis of patient-pharmacist conversations already conducted in a previous study. To achieve our aim, we undertook the following steps to show:

1. How well Discursis visually depicts episodes of pharmacist-patient engagement (Step 1)
2. Specific CAT strategies used by pharmacists that could be identified within the episodes of engagement (Step 2)
3. Differences in how pharmacist-patient speech patterns are displayed on Discursis plots for inpatient and outpatient settings (Step 3)

## Materials and methods

This is a descriptive study intended to demonstrate how Discursis software can be used to enhance or augment the qualitative analysis of previously analysed pharmacist-patient conversations. The video in S1 Video is the first of four short videos intended to provide an overview of Discursis.

### Previous research (basis of current study)

The first phase of this research studying pharmacist-patient communication took place at a 1000 bed teaching hospital that included multiple medical specialties within both outpatient and inpatient settings. We chose two different settings in order to observe whether there were different communication patterns associated with these different contexts. Twelve pharmacists had each engaged four patients for a total of 48 pharmacist-patient exchanges. Participating pharmacists were mostly women (83%), and about one-half were less than 30 years of age and had worked as a pharmacist for 10 years or less. About 56% of patients were male and older than 60 years of age. Study patients had been admitted to both inpatient areas (cardiology, emergency, geriatrics, general medicine, nephrology, neurology, oncology and surgery) and outpatient clinics (heart failure, infectious diseases and renal clinic).

The pharmacist-patient interactions were audio recorded, transcribed verbatim and analysed by selectively coding pharmacists’ dialogue for the five CAT strategies in pattern-based discourse analysis [39]. Analysis of the pharmacist-patient counselling sessions revealed that most pharmacists effectively used all five CAT communication strategies during medication counselling sessions as they adapted to patients’ conversational needs. Non-accommodation occurred when pharmacists spoke too quickly, used terms not understood by patients, and did not include patients in the initial, agenda-setting phase [21].

Further details about participant recruitment, inclusion criteria, data collection and analysis are described in the original completed study [21].

Research ethics approval was received from the Royal Brisbane and Women’s Hospital Human Research Ethics Committee (HREC/15/QRBW/433) and from the School of Pharmacy, The University of Queensland, Ethics Committee (2015/13). All participating pharmacists and patients provided written informed consent upon enrolment in the study.

### Discursis software

Discursis software [36, 37] is a validated, visual text analytic tool that accepts a text-based conversation transcript as input, and uses the Leximancer [40, 41] conceptual modelling algorithm to create a set of data-grounded concepts (see Table 2 for concept definition). This software is straightforward to operate where the user simply uploads their transcript files into the program. Then the software automatically applies a Bayesian statistical algorithm to determine the major conceptual content of the conversation. Each person’s turns in the conversation are represented by a set of concepts summarising their speech.

**Table 2:** Glossary of terms.

Term	Definition
Concept	Group of related words (identified by Leximancer) in a particular communication exchange; see video in S2 Video. for a more detailed description
Engagement, Episodes of	Points in time that indicate both speakers are contributing to the conversation (I.e. A two-way conversation); represented on Discursis as clusters of two-coloured or off/diagonal blocks
Leximancer	Software that uses a natural language processing algorithm to identify major concepts and themes taking place in a conversation; refer to video in S2 Video for a more detailed description
Low level engagement	Depicted by Discursis plots as conversations dominated by patterns of alternating, single coloured squares; very few or no clusters of off-diagonal blocks
Moderate-high level engagement	Depicted by Discursis plots as conversations containing multiple clusters of off-diagonal blocks throughout interaction
Off-diagonal block	Two-coloured block; represents a time when one speaker has picked up on another speaker’s concepts
One-way conversation	Where a conversation is dominated by one speaker, with little input from the other person
Recurrence plotting	Visualisation technique used to display and identify trends within time series data
Same-saying	One speaker repeats another speaker’s word (s)
Two-way conversation	Where turn taking happens and both speakers contribute to the conversation

Discursis has been specifically designed for analysing temporal aspects of communicative exchange. Its software uses an existing visualisation technique, called recurrence plotting, to display and identify trends over time [42]. The video in S2 Video demonstrates how Discursis uses the language program (Leximancer) and recurrence plotting to produce the plots.

**Fig 1:**
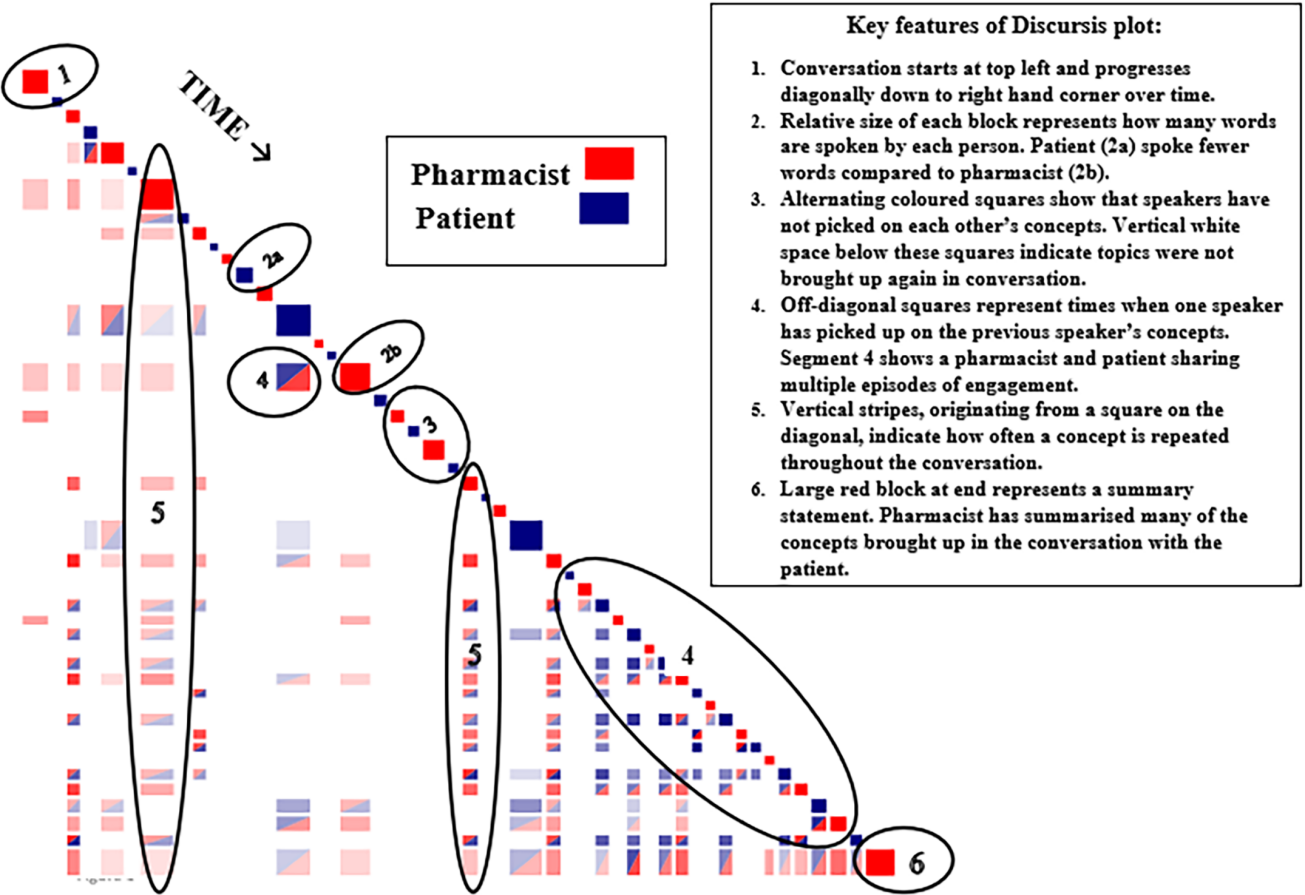
Key features of Discursis plots.

Discursis plots present conversations diagonally, turn-by-turn: to reveal the extent to which the speakers are using similar concepts to others, repeating their own concepts, or whether the topics are unrelated. If any two turns in a conversation contain similar concepts, then the corresponding vertical and horizontal intersection block (below the diagonal) is shaded in two colours to indicate conceptual similarity. Examples of these key features are included in Fig 1.

Several patterns on the Discursis plots reflect inter-speaker behaviours. Repetition of the same topic by a speaker or unrelated topics spoken by the speakers, appear as alternating, single coloured squares [38]. Vertical stripes extending downward from squares on the diagonal indicate where initial concepts are continued by a speaker over time. Horizontal stripes that originate from individuals’ turns and extend from right to left indicate a summary of concepts discussed up to that point in the exchange (Fig 1) [37].

In addition, the video in S3 Video explains the process of Discursis plot interpretation.

The Discursis plots are also interactive as the software allows the user to easily access the content of the conversation, thus verifying the exchanges at the level of each speakers’ words. Access to text is achieved by left clicking and dragging the mouse over the areas of interest and the software will magnify and reveal the dialogue adjacent to the plot squares. This allows immediate verification of the patterns observed (E.g. episode of engagement) and to investigate how well the speakers have stayed on topic. Visual examples of these magnified patterns with adjacent dialogue are shown and described in the Results section.

A glossary of terminology used in this paper can be found in Table 2.

### Process of pattern investigation

The 48 transcribed, audio recordings of pharmacist-patient interactions were uploaded to Discursis software to generate a visual representation for each conversation. All plots were examined in a step-wise approach to identify episodes of engagement or the absence of engagement between the speakers (Step 1), to examine in detail, the engagement episodes, to detect pharmacists’ application of the CAT strategies (Step 2), and to search for patterns associated with different practice settings (Step 3). Representative plots for each step were chosen for inclusion in this paper.

### Step 1—How well Discursis visually depicts episodes of pharmacist-patient engagement

**Fig 2:**
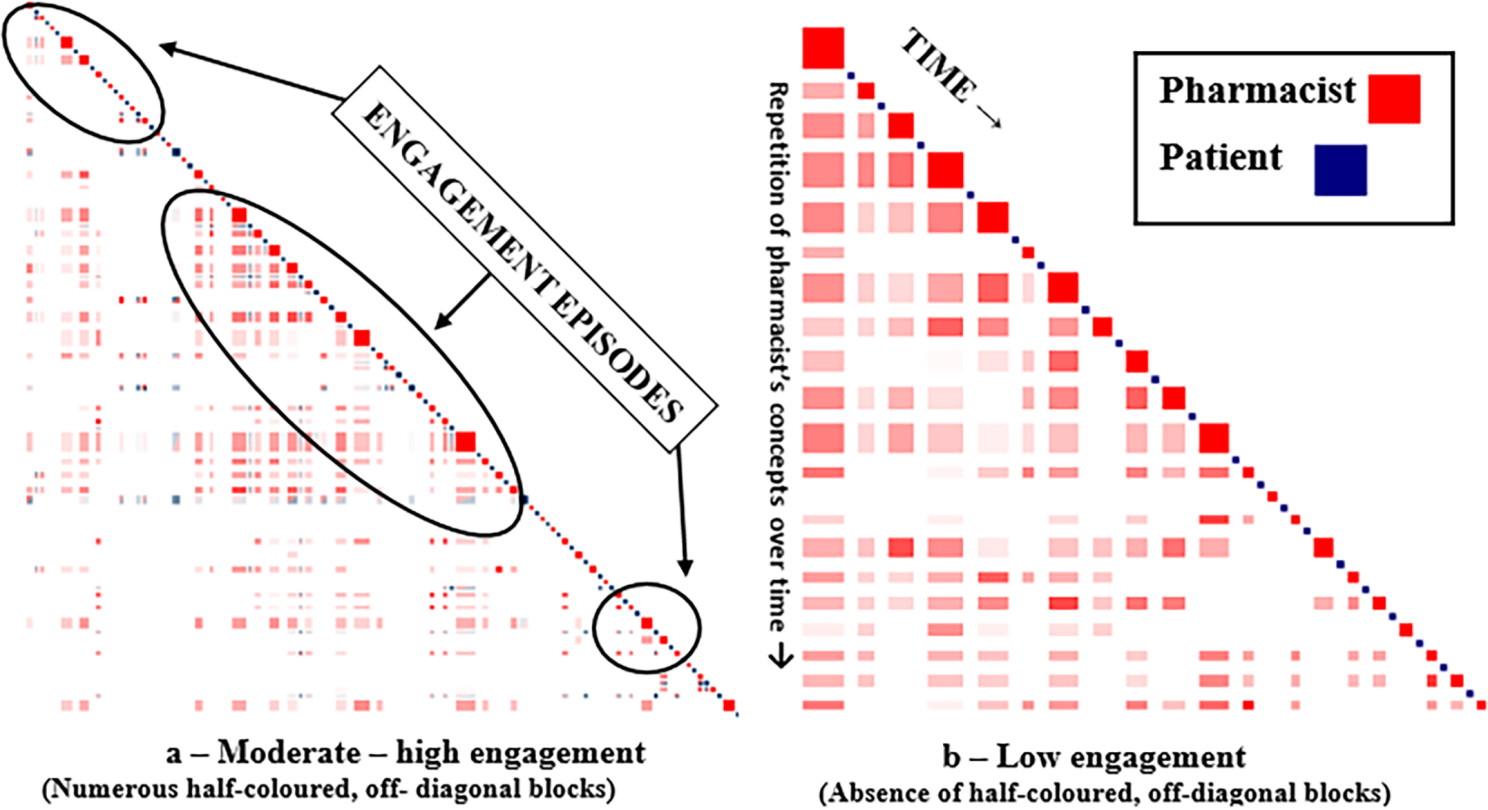
Inpatient setting—Moderate to high versus low engagement.

**Fig 3:**
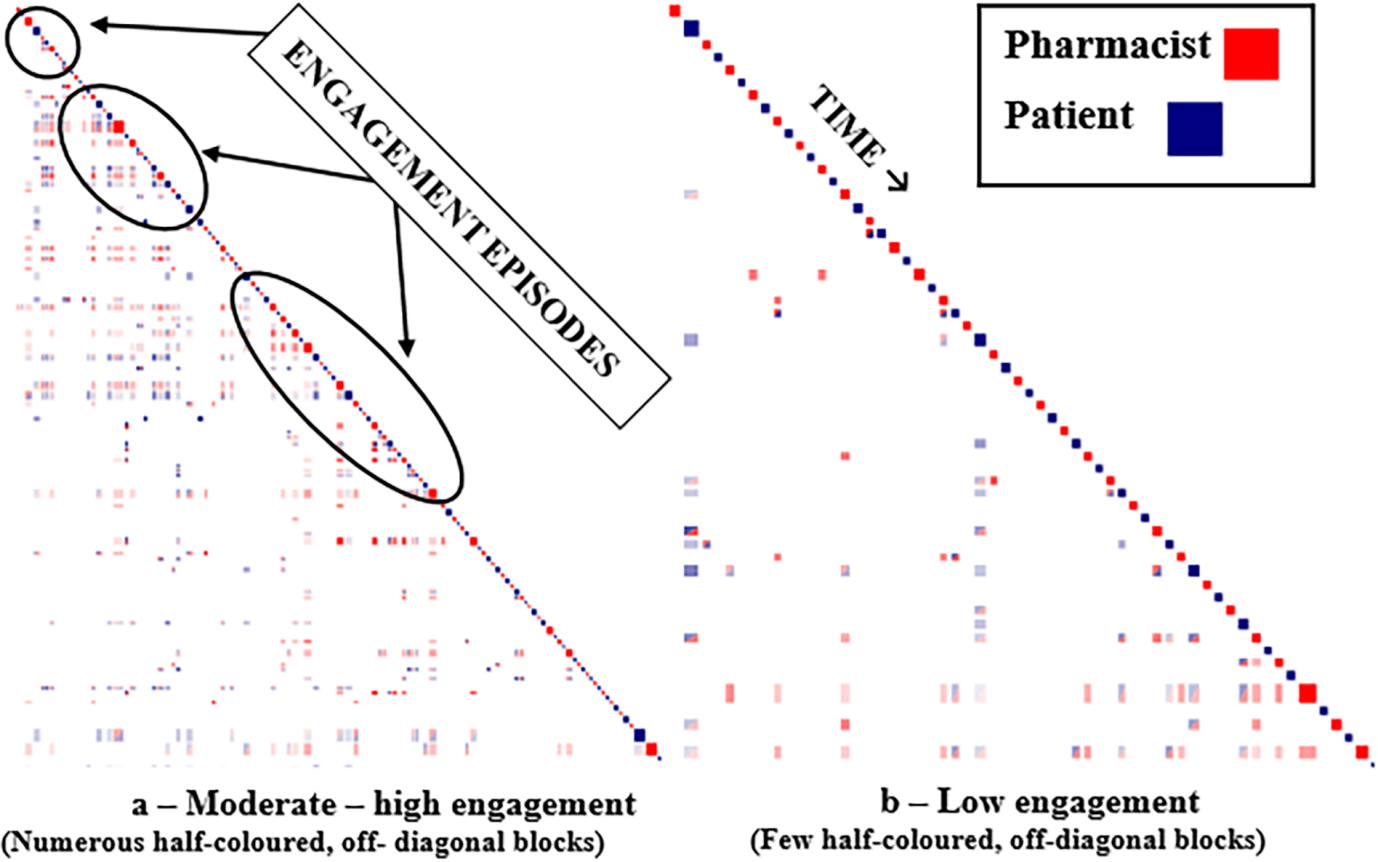
Outpatient setting—Moderate to high versus low engagement.

The first step in examining the plots was to determine the level of speaker engagement in each conversation. Although differences in levels of engagement are relative, our research group established indicators for moderate-high versus low levels of engagement. Moderate-high engagement was defined as conversations containing multiple clusters or episodes of recurring two-colour, off-diagonal blocks throughout the interaction (Refer to Figs 2 and 3 to illustrate these differences). This block pattern implies that speakers picked up on the context of each other’s conversation as their interaction progressed. Groupings of these events are considered times where speakers are engaged in a two-way conversation around specific concepts. In contrast, low levels of speaker engagement are typified by no or very few episodes of two-colour, off-diagonal blocks. Instead, these interactions are dominated by patterns of alternating, single coloured squares indicating that the speakers did not continue concepts from the previous speaker’s response. Representative plots of moderate-high and low levels of speaker engagement were chosen to demonstrate contrasts between the pharmacist-patient interactions. These plots were then reviewed by DA (Discursis developer and co-researcher) to verify their interpretation and engagement level designations. The video in S4 Video provides further details about the process of identifying episodes of engagement and how these can be verified while working in the program.

### Step 2—Specific CAT strategies used by pharmacists that could be identified within the episodes of engagement

**Fig 4:**
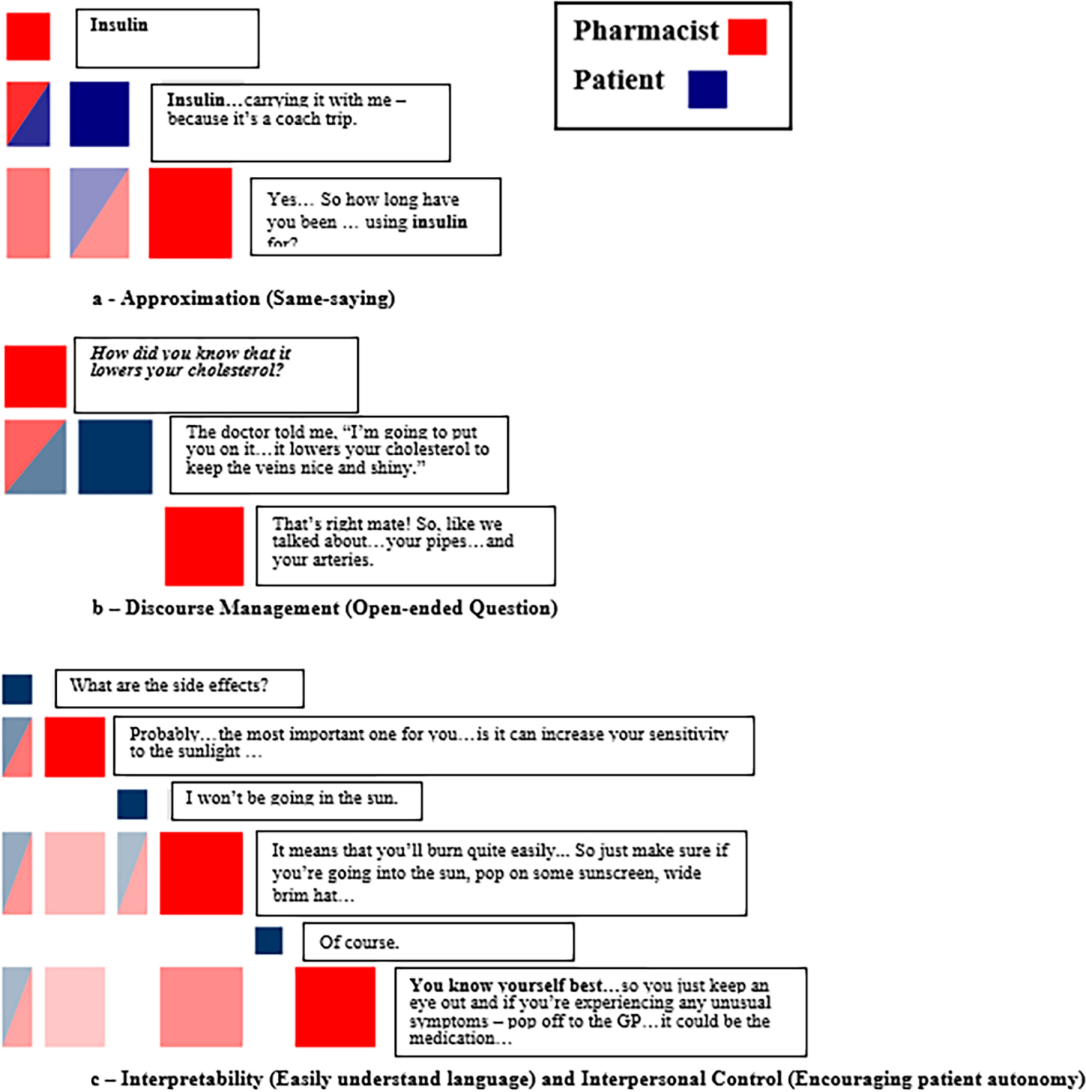
CAT strategies–Approximation, discourse management, interpretability and interpersonal control.

Detailed investigations of plots with moderate-high levels of engagement were conducted to determine whether specific CAT strategies used by pharmacists could be identified within the episodes of engagement (Fig 4). An assumption made was that moderate-high levels of engagement would more likely be representative of pharmacists accommodating as opposed to not accommodating patients’ conversational needs. Episodes of engagement and accommodative pharmacist behaviour within the groupings of off-diagonal blocks could be verified while working in the software program. This was done by left clicking and dragging the mouse over the engagement episode on the plot, which then expanded the area to reveal the actual, corresponding dialogue.

### Step 3—Differences in how pharmacist-patient speech patterns are displayed on Discursis plots for inpatient and outpatient settings

In addition to displaying episodes of speaker engagement, another important feature of Discursis is its ability to identify turn-taking dynamics between speakers indicating who speaks when and for how long. Plots from both inpatient and outpatient areas were compared. to determine whether differences in relative contributions to a conversation observed in pharmacist-patient exchanges could be attributed to the clinical setting in which they took place. For inpatient settings, most pharmacist-patient medication counselling sessions occurred around the time of patient discharge from hospital. At this transition, numerous healthcare professionals, including pharmacists, meet and speak with patients before they leave hospital. When medications are required by the patient, pharmacists either coordinate procurement with community pharmacists or provide a supply of discharge medications. In addition, all pharmacists provide a medication list and written information about new medications for patients at discharge. This medication list acts as a guide for conversations as pharmacists direct conversations typically in the order medications are presented on the list. Pharmacists discuss with patients which medications are current, new, and those to be discontinued. It is not uncommon for pharmacists to provide large amounts of information about the medications (e.g. drug name, rationale for use, dose and administration information, side effects and their management, strategies to promote adherence, and refill procurement directions). Typically, the medication counselling sessions are initiated by the pharmacist introducing themselves, stating the reason for their conversation, and providing a review of the written information. It was not unusual for pharmacists to be the dominant speaker during these sessions.

In outpatient or clinic settings, the type of conversation between pharmacists and patients tends to be about a specific medication issue or takes the form of a medication review where the pharmacists would ask patients to provide detailed information about their medication management. Pharmacists in outpatient settings also use medication lists to direct their conversations with patients; however, in these settings pharmacists often seek information and details about patients’ medication taking behaviour. Therefore, it is not uncommon for these outpatients to speak more than the patients from inpatient settings and reflects the outpatient context.

## Results

Discursis plots were generated from 48 pharmacist-patient conversations that took place in inpatient wards (36) and outpatient clinics (12).

### Step 1—How well Discursis visually depicts episodes of pharmacist-patient engagement

Moderate to high engagement plots are contrasted to low engagement plots in each of the inpatient and outpatient settings (Figs 2A and 3A).

Of the 48 Discursis plots, 40 conversations were designated as moderate-high pharmacist-patient engagement. There were 27 identified from inpatient and 11 in outpatient settings. Fig 2A from an inpatient ward includes the characteristic inpatient pattern of mostly larger red (pharmacist) squares, but also includes frequent clusters of two-colour, off-diagonal squares indicating engagement between the speakers. One large section of engagement (numerous off-diagonal square clusters) dominates the middle of the conversation where much of the discussion about the patient’s medications takes place. Certain concepts introduced by the pharmacist early in the conversation are carried throughout as depicted by the consistent reappearance of the squares in a vertical stripe. As well, the large red pharmacist square at the end of the conversation appears across the same horizontal line indicating that the pharmacist has included many of the previously discussed concepts in a summary statement to the patient.

Fig 3A depicts a Discursis plot from an outpatient clinic showing the larger blue (patient) squares typical for patient contributions in this type of conversation and from this setting. At the beginning of this exchange, the pharmacist and the patient engage in agenda-setting for the conversation. Concepts discussed in this early part of the conversation are carried throughout their interaction as indicated by the vertical stripes projected downward. This continuation is an indication that the conversation stayed on track for most of its course. As in the inpatient example, there is a clear delineation of concepts discussed in the mid-section of the exchange. Near the end of the conversation, the pharmacist (large red square) recaps several concepts already discussed with the patient as shown by the horizontal bar to its left containing numerous blue and red squares.

Eight low engagement Discursis plots could be distinguished from moderate-high engagement conversations by the absence or few occasions of two-colour, off-diagonal squares and the predominant pattern of alternating solid single coloured squares. There were seven inpatient and one outpatient conversations identified as low engagement. An example of a very low level of engagement between an inpatient hospital pharmacist and patient conversing at discharge is represented in Fig 2B. This plot contains no patterns indicative of engagement between the speakers. It is clear from the relatively large red blocks that the pharmacist is dominating the conversation. The repetition of the pharmacist’s speech (concepts) vertically and horizontally suggests they are mostly repeating themselves throughout the conversation. The pharmacist is staying on topic with little engagement from the patient who is only providing short responses or back-channelling utterances such as “hmm”. There are no blue vertical stripes stemming from early patient’s turns implying there was no partnership in setting the agenda for the conversation.

Fig 3B shows an exchange between an outpatient pharmacist and a patient where there are only a few occasions of engagement throughout the course of the conversation. Although there is lack of engagement in the early part of the exchange where the agenda- setting usually takes place, initial comments made by the patient about their medications are carried throughout the conversation as shown in the left-most downward vertical stripe. Frequent occurrences of white spaces underneath the diagonal sequence suggest multiple unrelated concepts have been raised by both speakers, but not necessarily continued throughout the conversation.

### Step 2—Specific CAT strategies used by pharmacists that could be identified within the episodes of engagement

Once initial patterns of engagement were identified, a more in-depth analysis of pharmacist-patient communication behaviour was conducted. Examples of moderate-high pharmacist-patient engagement were studied in detail to investigate how pharmacists applied CAT strategies to engage patients in their conversations. Pharmacists’ application of some of the CAT strategies were reflected in Discursis plots as episodes of engagement with the characteristic two-colour, off-diagonal patterns. Typically, these episodes represented times when pharmacists’ communication behaviours were accommodative of patients’ conversational needs. Accommodative examples were identified from four of the five CAT strategies, the exception being emotional expression.

Most of the approximation strategies (speech volume, tone, rate and accent) required review of audio recordings to be detected. However, Discursis plots demonstrated how pharmacists applied accommodative approximation through “same-saying” where they repeated patients’ phrases. These often appeared as shorter sequences of engagement with only one or two off-diagonal squares. Fig 4A is an example of a detailed plot showing pharmacist approximation to verify their understanding of patient’s words.

Accommodative discourse management strategies used by pharmacists were identified within episodes of pharmacist-patient engagement on Discursis plots (Fig 4B). In this plot, the details of the exchange show an inpatient pharmacist asking an open-ended question to a patient to ascertain his understanding of cholesterol medication.

Most pharmacists employed accommodative interpretability strategies by using easily understood terminology in their conversations with patients. Fig 4C shows a detailed plot of a discussion between a pharmacist and patient about managing a potential side effect. In this same exchange, the pharmacist also demonstrated accommodative interpersonal control by prefacing advice with the expression “you know yourself best” to encourage patient autonomy in making appropriate healthcare decisions.

The CAT strategy, emotional expression, was not identified in the segments of plots indicating moderate to high levels of pharmacist-patient engagement. (However, appropriate emotional expression was located within the original transcripts, audio recordings and observational notes that included descriptions of non-verbal behaviours such as facial expressions, nods, or physical contact).

### Step 3—Differences in how pharmacist-patient speech patterns are displayed on Discursis plots for inpatient and outpatient settings

**Fig 5:**
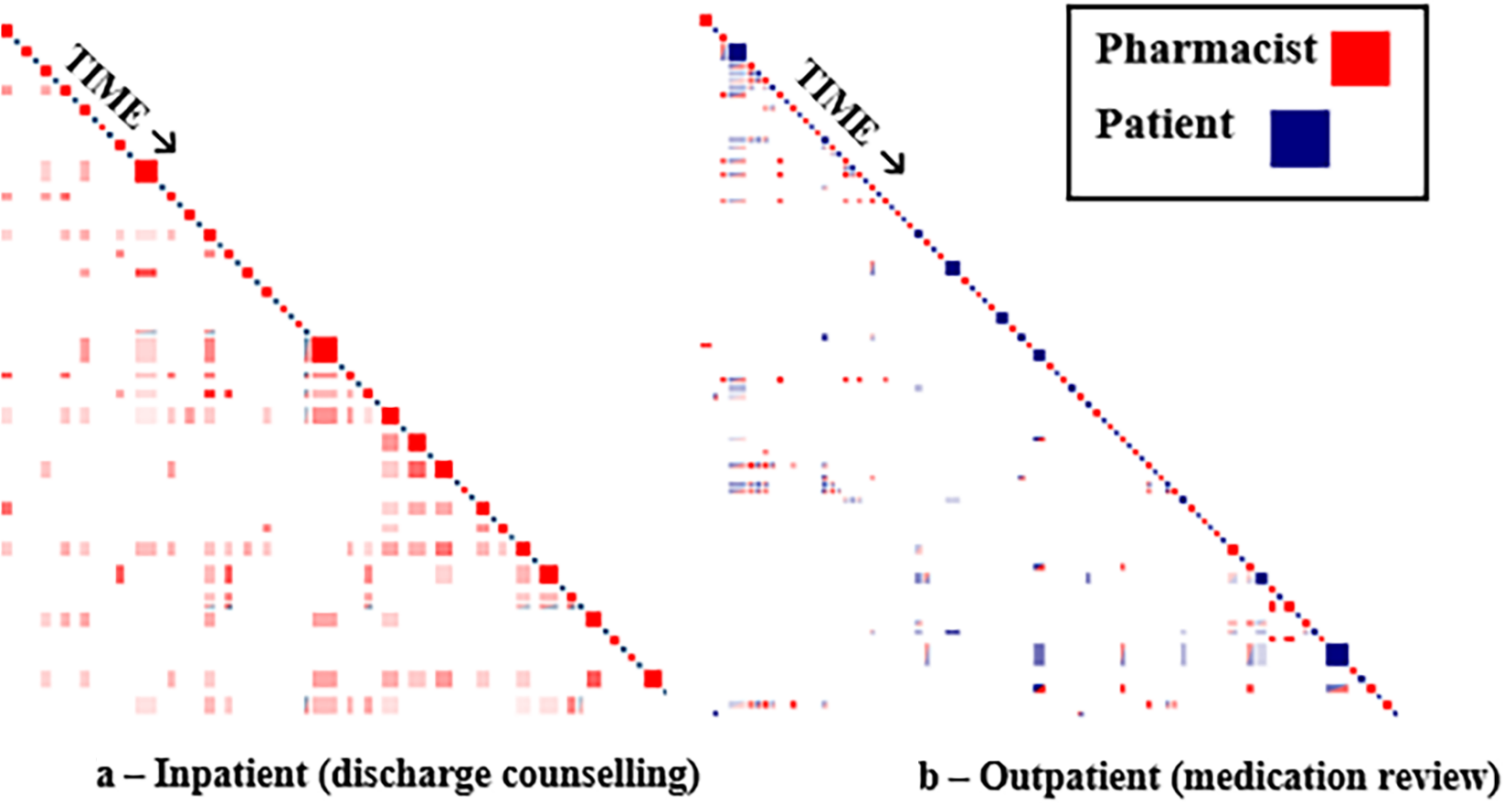
Pharmacist-patient medication counselling (inpatient versus outpatient settings).

Patterns reflecting pharmacist or patient speaker dominance were dependant on clinical setting in which their interaction took place. At the time of patients’ discharge from hospital, it was not uncommon for pharmacists to relay large amounts of medication information to inpatients as shown in the dominant squares of red (pharmacist) and smaller blue squares (patient) indicating fewer spoken words (Fig 5A).

Exchanges taking place in outpatient settings typically involved the pharmacists reviewing patients’ medication therapy by soliciting information from patients through questions and prompts. Fig 5B shows a plot of a pharmacist-patient exchange taking place in an outpatient clinic where the volume of conversation contributed by patients (blue) at least matches that of the pharmacists (red).

## Discussion

This study demonstrated that Discursis can enhance the qualitative interpretation of pharmacist-patient exchanges. The visualisation of conversations by Discursis allows users to quickly scan the plots and to view the relative contributions by each speaker as well as their level of engagement in the exchange. Although these features can be gleaned through qualitative analysis, Discursis facilitated their identification more efficiently and also permitted low and moderate-high engagement to be easily differentiated.

In addition to locating episodes of pharmacist-patient engagement, we were able to identify some specific CAT strategies used by pharmacists within these engagement episodes and to distinguish between interactions taking place in inpatient and outpatient settings. These findings corraborated the interpretation of the exchanges already analysed from audio recordings, transcripts and observational notes [21]. Discursis has been previously validated and used in other healthcare communication contexts where it has effectively been applied in the analyses of physician-patient consultations [38], conversations between dementia patients and residential care-givers [43], and healthcare provider-patient exchanges around disclosure of adverse events [44]. However, this study of pharmacist-patient communication is a novel use of Discursis.

Further discussion about how Discursis has contributed to the qualitative analysis is segregated based on Steps 1 to 3.

### Step 1—How well Discursis visually depicts episodes of pharmacist-patient engagement

Firstly, and of particular interest, was the software’s ability to represent different levels of pharmacist-patient engagement. Low and medium-high levels of engagement could be easily identified and distinguished on the Discursis plots. This feature will lend itself to future research where larger data sets of Discursis plots can be quickly scanned prior to detailed study.

Higher levels of engagement observed on Discursis typically signified a two-way conversation and were usually an indication of accommodative communication taking place between speakers. However, researchers have cautioned that strong engagement depicted by Discursis relays information about the level of speaker engagement, but does not indicate the content or relevance of the discussion. Angus et al. provided a Discursis example where the physician and patient were highly engaged in their conversation, but the topic was about sailing, and not the patient’s medical condition [38]. In contrast, pharmacists in this study did not allow conversations to digress and typically stayed on task throughout their exchanges with patients. This was confirmed by the process described in the Methods section to verify the dialogue corresponding to episodes of engagement (by highlighting these areas on the plots).

### Step 2—Specific CAT strategies used by pharmacists that could be identified within the episodes of engagement

The next step of the study involved “drilling down” to the original dialogue within the Discursis plots of moderate-high pharmacist-patient engagement. This detailed investigation offered numerous examples of accommodative use of CAT strategies by pharmacists for approximation, interpretability, discourse management and interpersonal control. No examples of the CAT strategy, emotional expression, could be detected in the Discursis engagement patterns. However, its absence is not entirely surprising as expressions of emotional support given by a pharmacist to a patient may not be concepts repeated back to the pharmacist by a patient. Therefore, these expressions would not be identified as related concepts by the software and would not be displayed as engagement (off-diagonal squares). Although emotional expression is a more abstract CAT strategy and could not be identified by Discursis within an engagement episode, its potential to establish positive pharmacist-patient exchanges and facilitate strong engagement with patients cannot be undervalued. The importance of accommodating patients’ emotional needs has been studied in physician communication research and has been positively associated with patient satisfaction and improved patient outcomes such as adherence to therapy and improved diabetic control [45, 46].

Examples of accommodative approximation as repetition of the previous speaker or “same-saying” were found in both isolated and larger episodes of engagement. Discursis patterns showed that both pharmacists and patients repeated portions of each other’s speech. Although both the pharmacists and patients were accommodating each other, they did so for different reasons. Pharmacists appeared to use “same-saying” to encourage patients to expand on the subject or clarify the patient’s meaning whereas patients repeated pharmacists’ speech not only to indicate their understanding of the topic, but also to seek clarification or ask a question. Research using Discursis and CAT to help analyse conversations during open disclosure about adverse events that occurred in hospital found that physicians usually used “same-saying” to reassure patients, however patients sometimes used “same-saying” negatively by repeating physician statements with sarcasm [44]. The negative use of “same-saying” was not observed in this study.

Accommodative interpretability and discourse management strategies utilised by pharmacists were readily identified within large segments of moderate-high engagement plots where pharmacists predominately used simple terms and phrasing in their discussions with patients and sought patient understanding by asking open-ended questions (Fig 4). Angus et al. inferred that within high level engagement exchanges where physicians spoke clearly and used easy-to-understand language, patients would be more likely to have a good understanding of their treatment plan [38]. On the other hand, the same researchers suggested that low physician-patient engagement as shown as alternating squares of colours meant that the patient left the consultation with little awareness of what the doctor said. It was postulated that the physician dominated the conversation and did not allow the patient enough time to take in the information and formulate responses [38]. Episodes of strong pharmacist-patient engagement, likely reflecting effective practitioner communication skills, are vital to ensure patients’ understanding of their medication therapy. Effective pharmacist-patient communication may, in future studies, be linked to improved patient outcomes such as medication adherence. Associations between treatment adherence and strong clinician-patient relationships have already been established in physician-patient communication literature [47].

The use of non-accommodative CAT strategies by pharmacists could not be detected within episodes of moderate-high engagement in the Discursis visualisations similar to findings by Angus et al. [38]. Instead, examples of non-accommodation were more readily found in exchanges classified as low level engagement such as Fig 2B. In fact, this exchange occurring early in the medication counselling session is likely to have had a negative effect on the remainder of the conversation. The tone and the content of this pharmacist’s speech heard in the audio recordings did not portray empathy, but conveyed judgement and frustration. There was little to encourage patient input in this conversation and it was not surprising that this conversation held no engagement between the pharmacist and patient. Interestingly, the pharmacist did not seem to recognise that their conversational method was ineffective and did not attempt to redirect their efforts to engage the patient. This pharmacist’s steadfast approach can be seen on the Discursis plot where the initial and subsequent vertical stripes are continuous throughout the conversation indicating that similar concepts were repeated multiple times. As well, the pharmacist dominated the conversation as seen by the larger red squares with only small blue squares representing the patient responses. Researchers observing similar Discursis patterns in physician-patient conversations have suggested that it is difficult to know how well patients have understood information when they have only provided abbreviated responses and there has been little engagement between the physician and patient [38]. Therefore, it is uncertain if this exchange provided any benefit to the patient’s understanding of their medications. As a caveat, it cannot be assumed that most or all situations of low patient engagement necessarily mean that the conversation has not been effective for the patient. For example, a patient wanting specific details about a medication may be content with receiving large volumes of information from the pharmacist and may only provide little input in terms of response. In this type of situation, parts of the interaction would likely be represented in a similar manner as in the example in Fig 2B. However, to determine that the patient preferred this type of exchange, an accommodative pharmacist would need to have discussed this with the patient, probably at an early point in the conversation. This agenda-setting phase would appear as an engagement episode on a Discursis plot and was not present in the exchange depicted in Fig 2B.

### Step 3—Differences in how pharmacist-patient speech patterns are displayed on Discursis plots for inpatient and outpatient settings

In the final step of the study, the setting of the conversations (inpatient versus outpatient) could be readily distinguished by the distribution and size of participants’ squares (turns) on the Discursis plots. For example, pharmacists were the dominant speakers for inpatient exchanges taking place before discharge from the hospital, whereas patients in outpatient settings provided more input during medication assessments.

Discursis has numerous practical and theoretical applications for future heath communication research and training. Its practical applications include: to provide a quick overview of exchanges between pharmacists (or other healthcare professionals) and patients to observe characteristic patterns of engagement between speakers, to identify dominant speakers and their ability to stay on task as well as good communication skills such as turn-taking and ensuring key concepts are summarised at the conversation conclusion. Discursis plots are easily generated from audio recorded transcripts, and therefore conducive as teaching tools for both students and practitioners to assess and develop their communication skills.

Reviewing Discursis plots for the presence or absence of engagement episodes is a logical first step prior to further qualitative analysis. This would allow researchers to use Discursis as a tool to conduct preliminary reviews of large data sets of clinician-patient conversations prior to identifying key areas for concentrated analysis.

Discursis was also amenable to the theoretical application of the communication theory, CAT, which allowed for a detailed examination and analysis of the individual episodes of engagement. By invoking CAT, this study provided theoretical rigor in a domain that is often not theory based, and therefore it makes a valuable contribution to health communication research.

There are limitations to this Discursis study of pharmacist-patient interactions. Current Discursis software is based on the transcriptions of the audio recordings, and does not, as yet, account for speakers’ pauses or non-verbal communication taking place. As well, Discursis software cannot identify emotional expression, an abstract CAT strategy, within an engagement episode. Understanding Discursis plots requires sufficient time and ample practice for most users to gain confidence in their ability to interpret the patterns accurately. However, once this is achieved, there is much potential for Discursis to enrich the qualitative analysis of communication research. Another potential limitation was the self-selection of highly motivated pharmacists, who chose to be part of the original pharmacist-patient communication study. This may have resulted in fewer examples of poor communication from which to select for Discursis analysis, and in turn, may limit the transferability of positive results.

## Conclusion

Discursis software was an effective and efficient technology to enhance the qualitative analysis of pharmacist-patient conversations by providing visual representations of the interactions. Characteristic patterns displayed by Discursis showed the relative contributions made by each speaker, the extent of pharmacist-patient engagement, and how well the conversation remained on topic, all aspects of an effective exchange. Discursis has value as an adjunct to analysis in qualitative research as well as a teaching tool in communication skills training for both students and practitioners.

## Supporting information

S1 Video What is Discursis? (Part 1). (MP4)

S2 Video How does Discursis work? (Part 2). (MP4)

S3 Video How to Interpret Discursis plots (Part 3). (MP4)

S4 Video Episodes of engagement (Part 4). (MP4)
